# Prevalence, associated factors and perinatal outcomes of antepartum depression in Ibadan Nigeria

**DOI:** 10.1186/s12884-022-04549-7

**Published:** 2022-03-18

**Authors:** Ikeola A. Adeoye, Abiodun Sogbesan, Oluyomi Esan

**Affiliations:** 1 Department of Epidemiology and Medical Statistics, College of Medicine, University of Ibadan, Ibadan, Nigeria; 2 Consortium for Advanced Research in Africa (CARTA), Nairobi, Kenya; 3 Department of Psychiatry, College of Medicine, University of Ibadan, Ibadan, Nigeria

**Keywords:** Antepartum depression, Maternal health, Mental health, Birth outcomes, Nigeria

## Abstract

**Background:**

Antepartum depression is the most common mental health disorder in pregnancy and it is also a risk factor for adverse perinatal outcomes. Low and middle income countries like Nigeria bear a higher burden of antepartum depression compared with high income countries. Prioritizing mental health issues among pregnant women is crucial to achieving the Sustainable Development Goals. We determined the prevalence, associated factors and perinatal outcomes of antepartum depression among pregnant women in Ibadan, Nigeria.

**Methods:**

A prospective cohort study was conducted among 1745 pregnant women enrolled early in pregnancy (≤ 20 weeks) at four comprehensive obstetric facilities within Ibadan metropolis. Antepartum depression was ascertained during the third trimester using the Edinburg Postpartum Depression Scale ≥ 12. The primary exposure was antepartum depression and the outcome variables were the perinatal outcomes. The associated factors assessed included sociodemographic, obstetric, psychological, and lifestyle characteristics. Bi-variate logistic and Poisson regression analyses were used to assess the factors and relative risk for perinatal outcomes of antepartum depression.

**Results:**

The prevalence of antepartum depression was 14.1%. The significant factors associated with APD after adjusting for confounders were: high income (≥ 20, 000) which was protective (AOR) = 0.59; 95% CI: (0.40 – 0.88); *p*-value: 0.010] and perceived stress increased the odds of APD in a monotonic fashion: moderate stress [AOR = 2.39; 95% CI: (1.01 – 5.7); *p*-value: 0.047], high stress [AOR = 6.43; 95% CI: (2.28 – 18.2); *p*-value: < 0.001]. Preterm delivery was the only significant perinatal outcome [Relative Risk (RR) = 1.66; 95% CI (1.14 – 2.39); *p*-value =  0.007]. Depression did not increase the risk of having low birth weight babies (*p* = 0.513), macrosomia (*p* = 0.894), birth asphyxia (*p* = 0.317), and caesarean section (*p* = 0.298).

**Conclusions:**

APD was prevalent among our study population. The significant factors identified in this study can be targeted to reduce the occurrence of APD among pregnant women in Nigeria through appropriate social and public health interventions which include APD screening, counselling, and the provision of emotional support for pregnant women during antenatal care.

## Background

The World Health Organization defines mental health as “a state of well-being in which every individual realizes his or her own potential, can cope with the normal stresses of life, can work productively and fruitfully, and is able to make a contribution to her community” [1]. Conversely, mental disorder are disturbances of thought, emotion, behaviour, and relationships with others that lead to substantial suffering and functional impairment in one or more major life activities [2]. Maternal mental health disorders have become a significant public health concern because of their harmful effects on both mothers and their infants [3, 4]. Worldwide, about 300 million people are affected by depression and it is one of the major causes of disability [5]. Antepartum depression (APD) is a non-psychotic mood disorder characterized by a decreased interest or pleasure in almost all activities, significant weight loss, disturbed sleep, fatigue, loss of appetite, feeling of hopelessness, lowered self-esteem, as well as a diminished ability to think or concentrate, occurring during pregnancy [6]. Even though, depression is the most common mental disorder of pregnancy it is often neglected in low and middle-income countries, where the burden is greater compared with higher income countries [7]. According to the WHO the prevalence of APD in LMIC ranges from 12 to 42% [4]. The pooled prevalence of APD in sub-Saharan Africa is 26.3% [7] while it ranges from 8.3 to 26.6% in Nigeria [8–11] Antepartum depression can interfere with the woman’s bio-social adjustment to pregnancy, daily activities and functioning, as well as spousal, family and community relations [12].

The factors associated with APD can be broadly classified into sociodemographic, obstetric, lifestyle and psychosocial factors. The psychosocial factors include those triggered by marital disharmony, domestic violence, stress, lack of social support. Poor lifestyle factors like tobacco use, alcohol consumption have also been implicated. Additionally, previous studies have reported low socioeconomic status [13, 14], low level of education [14] young age [11] sleep deprivation [15] lack of support from family and loved ones [13] lack of social support and partner support [7] obesity [16]), unplanned pregnancies [11] exposure to cigarette [7] history of obstetric complications, history of depression and common mental disorder [7, 14]. Exposure to domestic violence, history of physical and sexual abuse, partner neglect, elevated stress levels, relationship conflicts are also correlates of APD [13, 17].

Notably, antepartum depression has been associated with adverse maternal and perinatal outcomes which include preeclampsia, preterm delivery, low birth weight, smaller head circumference, lower Apgar Scores and a higher incidence of childhood behavioural disorders later in life [18–21]. APD is also a critical risk factor for postpartum depression which hinders child development, mother and infant interaction and the family life [22]. However, only a few studies in LMIC [23, 24] have investigated APD—associated perinatal outcomes because of the limitation of study design employed. Cross-sectional studies only gives a snapshot of the evidence, whereas retrospective studies are limited by recall bias or incomplete data. Longitudinal studies that follow the same cohort of women during pregnancy right into the post-partum period have the advantage of portraying the associated perinatal events. Hence, longitudinal studies are essential for capturing APD associated perinatal outcomes, unfortunately these are uncommon in sub-Saharan Africa including Nigeria. Bindt and colleagues are one of the few researchers that have explored the pregnancy and child outcomes of depression in Africa using longitudinal studies [12, 22, 25, 26]. Their study, among a cohort of pregnant women in Ghana and Cote D’Ivoire, did not show any association with perinatal outcomes because it was conducted among women with a low obstetric risk [25]. Although, they found an association between APD and the disruption of daily function. Recently, Mac Girth and his coworkers (2020), investigated the associations between APD and infant and developmental outcomes using a South African birth cohort [27]. Therefore, we investigated the prevalence, associated factors and perinatal outcomes of antepartum depression among a cohort of pregnant women in Ibadan, Nigeria using the Ibadan Pregnancy Cohort Study.

## Methods

### Study design and participants

**Fig. 1:**
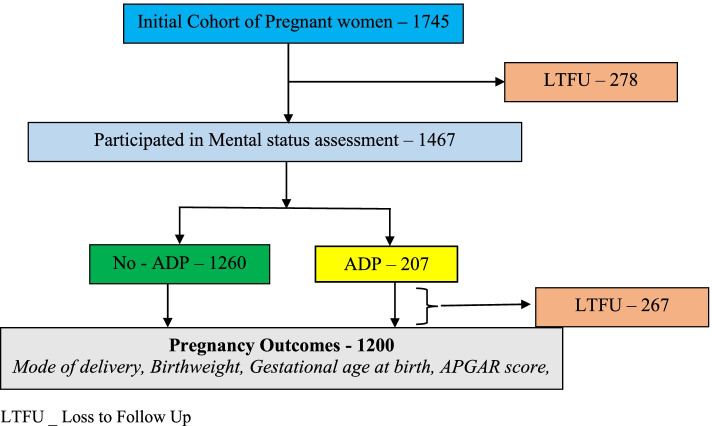
Flow chart of study participants from recruitment until delivery

This was a component of a prospective cohort study – The Ibadan Pregnancy Cohort Study (IbPCS) which primarily aimed at assessing the associations of maternal obesity and lifestyle factors on pregnancy and postpartum outcomes among women and their offspring in Ibadan. The study setting was Ibadan, the capital city of Oyo State, southwest, Nigeria. We recruited women in early pregnancy (≤ 20 weeks) from the four comprehensive obstetric facilities which are the main referral centers for complex obstetric cases within the Ibadan metropolis. These facilities were the University College Hospital, Adeoyo Maternity Teaching Hospital, Jericho Specialist Hospital and Saint Mary Catholic Hospital, Ibadan. The eligibility criteria included women ≤ 20 weeks’ gestation, aged ≥ 18 years, women without severe medical complications. After enrollment, participants were followed up and assessed at different time points: second, third trimesters, and delivery. At recruitment, the women’s baseline characteristics which included sociodemographic, lifestyle characteristics, obstetric and medical histories were ascertained. Maternal mental health status namely Depression, perceived stress, binge eating, marital disharmony (assessed by the occurrence of intimate partner violence during pregnancy) was examined in the third trimester. Maternal and birth outcomes were ascertained at delivery. The details of the methodology (study population, sample size and selection) has been reported is reported elsewhere [28]. The study protocol was approved by UI/UCH IRB Ethics and Research Committee and the Oyo State Ethical board. The study was conducted according to the declaration of Helsinki. The flow chart of the study is shown in Fig. 1. In all, one thousand seven hundred and forth-five women were recruited at baseline. Only 1467 were available for mental health assessment during the 3^rd^ trimester and 1200 at delivery.

### Measures

Antepartum depression was ascertained during the third trimester using the Edinburgh Postnatal Depression Scale (EPDS). The EPDS is the commonest tool for screening for depression among pregnant women and postpartum mothers. The EPDS is a valid and useful instrument in screening for depression in late pregnancy among Nigerian women [29] with EPDS ≥ 12 for major depression (sensitivity = 1.000, specificity = 0.961, diagnostic likelihood ratio for a positive result = 25.641). A 10—item instrument with 4—Likert scale responses from 0 to 3. The total score is derived from the summation of the 10 responses to give a value ranging from 0 to 30. A score ≥ 12 indicates antepartum depression [30]. Intimate Partner Violence was assessed using the Hurt, Insult, Threaten and Scream (HITS) tool for Intimate Partner Violence Screening developed by Kevin et al., 1998. It is a simple and fast tool that has 5 items with a 5-point scale (never, rarely, sometimes, fairly often and frequently) score from 1–5 for each item. The total scoring of this tool ranges from 1 to 20 and a score greater than 10 is regarded as been positive for Intimate partner violence [31].

**Fig. 2:**
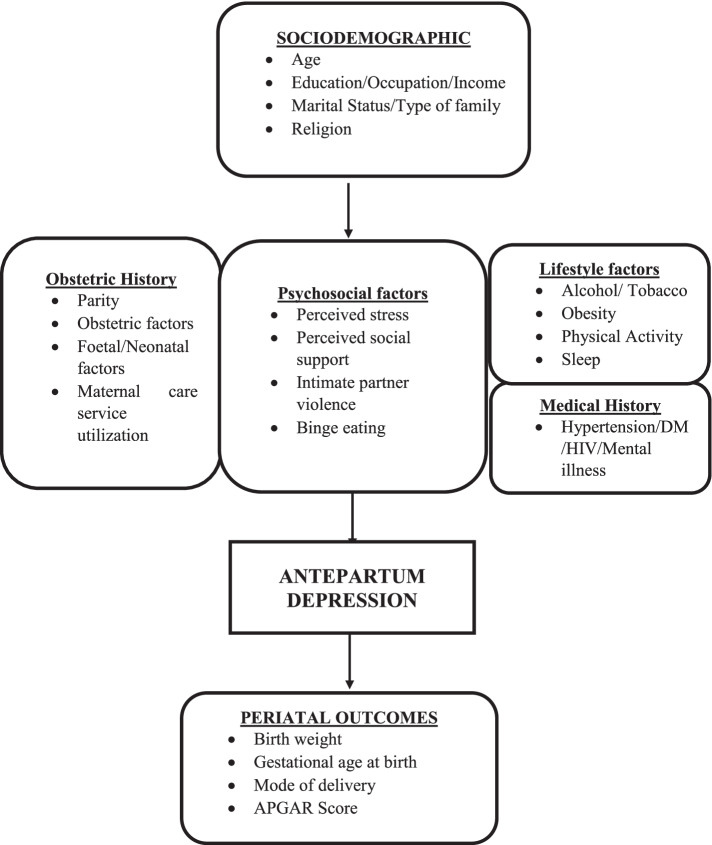
Conceptual framework for antepartum depression

Perceived stress was measured using the Perceived Stress Scale (PSS) [32]. The scale comprises 10 questions that represent an individual’s perceived stress. Each question has 4 multiple choice answers giving a maximum score of 40, individual scores ranged from 0—40. Scores are categorized into low stress (0–13), moderate stress (14–26) and high stress (27–40). Binge eating was defined in this study as overeating to relieve stress even when you are not hungry with the following responses: 3—yes, quite often; 2—sometimes; 1 – hardly ever; 0 – never. The explanatory variables examined in this study included socio-demographic, obstetric, psychological and lifestyle factors with their relationship with the study outcomes – APD and perinatal outcomes are shown in the conceptual framework in Fig. 2.

### Statistical analysis

Statistical analysis was performed using STATA version 13. Bivariate analysis was conducted to characterize the differences in the proportion of the women with or without antepartum depression using the chi-square test, according to their socio-demographic, maternal, obstetric, lifestyle and psychosocial actors and infant characteristics. T-test was also used to assess the differences in the means of continuous variables. For the multivariate analysis; the dependent variable was antepartum depression and the independent factors derived from the conceptual framework – background, proximate and clinical factors which were fitted into the models one after the other. Bivariate logistic regression output: Unadjusted and Adjusted Odds Ratios, 95% confidence intervals and *p*-values were reported. Factors included in the final model were those found significant (*p* < 0.05) at the bivariate level. The incidence of perinatal outcomes was obtained and also examined for significant association using Poisson regression with IRR and 95% confidence intervals. The dependent variables were low birth weight, macrosomia, birth asphyxia, preterm delivery and caesarean section.

## Results

### Socio-demographic, maternal, obstetric and psychosocial characteristics

**Fig. 3:**
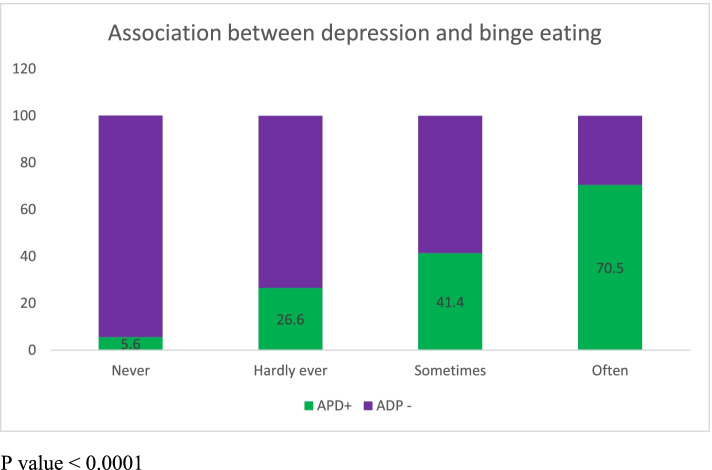
Association between depression and binge eating

The socio-demographic, maternal, obstetric, lifestyle, psychosocial and infant characteristics of the participants by their depressive status are presented in Tables 1 and 2. The mean age was 29.3 ± 5.5 years and 30.0 ± 5.3 years among women with APD and the controls respectively. Although the women with APD were younger, the mean age of the two groups was not significantly different (*p* = 0.081). The prevalence of antepartum depression in this study was 14.1%. The prevalence of APD was significantly higher among the unmarried women (21.7%), unemployed women (19.4%), lower-income earners (17.0%), and poorer women (16.1%). Additionally, women with a history of previous induced abortion (20.0%), currently experience of intimate partner violence (40.0%) also had a higher occurrence of APD. Perceived stress had a dose–response relationship with APD: low stress (6.4%), moderate stress (15.2%), and high stress level (28.7%). There was a significant association (*p* = 0.001) between binge eating and antepartum depression (Fig. 3).

**Table 1:** Socio-demographic characteristics of participants by their depressive status in Ibadan, Nigeria

Characteristics	Total (1467)	Antepartum Depression		Unadjusted OR **(95% CI)**	*P*-value
Overall		**Yes** **207 (14.1)**	**No** 1260 (85.9)		
**Age (Years)**					
< 20	255 (1.70)	5 (20.00)	20 (80.00)	1	
20—29	697 (47.5)	103 (14.78)	594 (85.22)	0.693 (0.255, 1.889)	0.474
30—39	693 (47.2)	91 (13.13)	602 (86.87)	0.604 (0.221, 1.651)	0.326
40 +	52 (3.6)	8 (15.38)	44 (84.62)	0.727 (0.211, 2.503)	0.614
**Mean Age ± SD**	29.9 ±	29.3 ± 5.47	30.0 ± 5.30		0.081
**Marital Status**					
Single	83 (5.70)	18 (21.69)	65 (78.31)	1	
Currently Married	1384 (94.3)	189 (13.66)	1195 (86.34)	0.571 (0.331, 0.984)	*0.044
**Family Type**					
Monogamous	1270 (93.2)	181 (14.25)	1089 (85.75)	1	
Polygamous	93 (6.8)	12 (12.90)	81 (87.10)	0.891 (0.476, 1.667)	0.719
**Maternal Education**					
Primary or less	40 (2.70)	6 (15.00)	34 (85.00)	1	
Secondary	417 (28.5)	73 (17.51)	344 (82.49)	1.20 (0.487, 2.969)	0.689
Tertiary	1006 (68.8)	128 (12.72)	878 (87.28)	0.826 (0.340, 2.007)	0.673
**Employment Status**					
Unemployed	170 (11.6)	33 (19.41)	137 (80.59)	1	
Employed	1297 (88.4)	174 (13.42)	1123 (86.58)	0.643 (0.426, 0.971)	*0.036
**Religion**					
Christianity	841 (57.7)	113 (13.44)	728 (86.56)	1	
Islam	617 (42.3)	93 (15.07)	524 (84.93)	1.143 (0.850, 1.538)	0.376
**Ethnicity**					
Non-Yoruba	152 (10.4)	20 (13.16)	132 (86.84)	1	
Yoruba	1312 (89.6)	186 (14.18)	1126 (85.82)	1.090 (0.664, 1.789)	0.732
**Income**					
Less than 20,000	489 (38.1)	83 (16.97)	406 (83.03)	1	
20,000–99,999	706 (55.1)	83 (11.76)	623 (88.24)	0.65 (0.46—0.91)	0.011
100,000 and above	87 (6.8)	9 (10.34)	78 (89.66)	0.56 (0.27 – 1.17)	0.124
**Wealth Index**					
Poor	476 (32.5)	80 (16.81)	396 (83.19)	1	
Middle	489 (33.3)	68 (13.91)	421 (86.09)	0.799 (0.562, 1.136)	0.212
Rich	502 (34.2)	59 (11.75)	443 (85.89)	0.659 (0.459, 0.947)	*0.024

**Table 2:** Maternal, obstetric, lifestyle, psychosocial and infant characteristics of participant by their depressive status in Ibadan, Nigeria

Characteristics	Total	Antepartum Depression		Unadjusted OR (95% CI)	*P*-value
		**Yes**	**No**		
**Gravidity**					
Primigravida	481 (32.9)	62 (12.89)	419 (87.11)	1	
2–4	822 (56.3)	116 (14.11)	706 (85.89)	0.999 (0.739, 1.352)	0.997
5 and above	158 (14.0)	29 (18.35)	129 (81.65)	0.863 (0.415, 1.797)	0.694
**Parity**					
Nullipara	641 (43.9)	91 (14.20)	550 (85 .80)	1	
1–3	747 (51.2)	106 (14.19)	641 (85.81)	1.110 (0.797, 1.546)	0.535
4 and above	72 (4.9)	9 (12.50)	63 (87.50)	1.519 (0.937, 2.463)	0.090
***Obstetric history***					
Previous Miscarriage					
Yes	321 (30.4)	50 (15.58)	271 (84.42)	1.048 (0.729, 1.508)	0.799
No	735 (69.6)	110 (14.97)	625 (85.03)	1	
Previous Induced Abortion					
Yes	245 (25.4)	49 (20.00)	196 (80.00)	1.688 (1.153, 2.472)	*0.007
No	773 (74.6)	93 (12.90)	628 (87.10)		
Previous Still birth					
Yes	133 (13.7)	22(16.54)	111 (83.46)	1.170 (0.711, 1.918)	0.539
No	841 (86.3)	122 (14.51)	719 (85.49)		
Previous Caesarean Section					
Yes	215 (21.8)	24 (11.16)	191 (88.84)	0.677 (0.425, 1.080)	0.101
No	773 (78.2)	121 (15.65)	652 (84.35)		
***Lifestyle characteristics***					
Tobacco exposure					
Yes	52 (3.5)	7 (13.46)	45 (87.80)	0.945 (0.420, 2.125)	0.089
No	1415 (96.5)	200 (14.13)	1215 (86.1)	1	
Alcohol Consumption					
Yes	191 (13.0)	30 (15.7)	161 (84.3)	1.157 (0.760, 1.762)	0.680
No	1276 (87.0)	177 (13.9)	1099 (86.1)	1	
Maternal Obesity					
Yes	279 (19.6)	46 (16.49)	233 (83.51)	1.226 (0.857, 1.753)	0.265
No	1146 (80.4)	159 (13.87)	987 (86.13)	1	
Duration of moderate intensity exercise (± SD)	26.3 (± 22.9)	25.4 (± 23.3)	26.4 (± 22.9)	1.00 (0.991, 1.005)	0.546
Duration of sleep (± SD)	8.03 (± 0.65)	8.01 (± 1.78)	8.01 (± 1.67)	1.00 (0.91, 1.09)	0.964
***Psychosocial factors***					
Intimate Partner Violence					
Yes	10 (0.7)	4 (40.00)	6 (60.00)	4.11 (1.15, 14.7)	0.029
No	1457 (99.3)	203 (13.93)	1254 (86.07)		
Perceived Stress					
Low	144 (10.8)	10 (6.94)	134 (93.06)	1	
Moderate	1106 (82.7)	168 (15.19)	938 (84.81)	2.440 (1.031, 5.776)	*0.042
High	87 (6.5)	25 (28.74)	62 (71.26)	6.129 (2.155, 17.431)	*0.001
Chronic Medical Illness					
Yes	149 (10.2)	24 (16.11)	125 (83.89)	1.191 (0.749, 1.893)	0.740
No	1318 (89.8)	183 (13.88)	1135 (86.12)		
***Infant factors***					
Sex					
Male	631 (52.7)	95 (15.1)	536 (85.9)	1.10 (0.802 – 1.53)	0.531
Female	566 (47.3)	78 (13.8)	488 (86.2)	1	
Mean Birth weight (kg, mean ± SD)	3.10 (± 0.53)	3.07 (± 0.55)	3.11 (± 0.52)		0.473
Mean Gestational age (weeks, mean ± SD)	38.9 (± 2.79)	38.6 (± 3.52)	38.9 (± 2.79)		0.046

### Factors associated with antepartum depression

Unadjusted logistic model, showed that marital status, employment status, income and wealth status were the significant sociodemographic factors associated with antepartum depression. Specifically, being currently married [unadjusted odd’s ratio (UOR) = 0.57; 95% confidence interval CI: (0.33 – 0.98); *p*-value: 0.044], being employed [UOR = 0.57; 95% CI: (0.33 – 0.98); *p*-value: 0.044], higher income [UOR = 0.65; 95% CI: (0.46 – 0.91); *p*-value: 0.011], being rich [UOR = 0.66; 95% CI: (0.46 – 0.95); *p*-value: 0.024], lowered the odds of APD. Conversely, history of previous induced abortion increased the odds of APD by 69% compared to women who had never procured abortion [UOR = 1.69; 95% CI: (1.15 – 2.47); *p*-value: 0.007]. The odds of APD also increased monotonically with the level of stress perceived by the women: moderate stress [UOR = 2.44; 95% CI: (1.03 – 5.78); *p*-value: 0.042], high stress [UOR = 6.13; 95% CI: (2.16 – 17.4); *p*-value: 0.001].

In the adjusted model, factors associated with APD are shown in Table 3, and only income and perceived stress remained significant. Particularly, earning a higher income (≥ 20, 000) was protective of antepartum depression by 41% [AOR = 0.59; 95% CI: (0.40 – 0.88); *p*-value: 0.010] after adjusting for other factors. Perceived stress still remained a significant factor in a monotonic fashion after adjusting for other factors. Moderate stress [AOR = 2.39; 95% CI: (1.01 – 5.7); *p*-value: 0.047], high stress [AOR = 6.43; 95% CI: (2.28 – 18.2); *p*-value: < 0.001].

**Table 3:** Adjusted OR and 95% confidence interval of factors associated with Antepartum haemorrhage in Ibadan, Nigeria

Variables	Adjusted OR (95% CI)	*P*-value
**Marital Status**		
Single	1.00	
Currently Married	0.66 (0.25 – 1.72)	0.393
**Income**		
< 20,000	1.00	
≥ 20,000	0.59 (0.40 – 0.88)	0.010*
**Previous Induced Abortion**		
Yes	1.49 (0.97 – 2.29)	0.065
No	1.00	
**Intimate Partner Violence**		
Yes	3.21 (0.73 – 14.1)	0.124
No	1.00	
**Perceived Stress**		
Low	1.00	
Moderate	2.39 (1.01 – 5.66)	0.047*
High	6.43 (2.28 – 18.2)	< 0.001*

### Perinatal outcomes

The incidence and relative risk (95% CI) of perinatal outcomes among women with antepartum depression in Ibadan are shown in Table 4. The incidence of LBW (6.4%), macrosomia (5.8%), preterm delivery (21.7%), birth asphyxia at one minute (12.3%) and caesarean section. (28.8%) among women with APD. Preterm delivery was the only perinatal outcome with a significantly higher relative risk among depressed [Relative Risk (RR) = 1.66; 95% CI (1.14 – 2.39); *p*-value = 0.007] compared to women that are not depressed. Depression did not increase the risk of having low birth weight babies (*p* = 0.513), macrosomia (*p* = 0.894), birth asphyxia at 1 min (*p* = 0.317), and caesarean section (*p* = 0.298).

**Table 4:** Incidence and relative risk (95% CI) of perinatal outcomes among women with antepartum depression in Ibadan, Nigeria

Perinatal Outcomes	Depression	No Depression	Relative Risk 95% CI	*P*-value
**Low birth weight**				
Cases (Incidence)	**10 (6.4)**	76 (7.9)	0.81 (0.43 – 1.53)	0.513
Non—cases	146 (93.6)	275 (92.1)	1.00	
**Macrosomia**				
Cases (Incidence)	**9 (5.8)**	58 (6.0)	0.95 (0.48 – 1.88)	0.894
Non—cases	147 (94.2)	902 (94.0)	1.00	
**Preterm delivery**				
Cases (Incidence)	**36 (21.7)**	130 (13.0)	1.66 (1.14 – 2.39)	0.007
Non—cases	130 (78.3)	864 (87.0)	1.00	
**APGAR Score at 1 min**				
Cases (Incidence)	**13 (12.3)**	112 (16.1)	0.76 (0.44 – 1.30)	0.317
Non—cases	93 (87.7)	586 (83.9)	1.00	
**Caesarean section**				
Cases	**51 (28.8)**	345(32.8)	0.88 (0.69 – 1.12)	0.298
Non—cases	126(71.2)	708 (67.2)	1.00	-

## Discussion

Mental health is an integral part of the Sustainable Development Goals as it calls on all countries to reduce premature mortality from non-communicable diseases by a third through prevention and treatment as well as the promotion of mental health and well-being by 2030 [2]. Maternal mental health is also crucial for optimizing pregnancy and neonatal outcomes as well as ensuring long term health and functioning. In this study we investigated the prevalence, associated factors and the perinatal outcomes of antepartum depression using a prospective cohort study design among pregnant women in Ibadan, Nigeria. The prospective design enabled us to investigate perinatal outcomes among our study population. Previous studies have been limited in investigating the perinatal outcomes associated with maternal mental concerns because of the study design that was utilized, mostly cross-sectional [9, 11, 33]. Notably, the prevalence of antepartum depression in this study was 14.1%. This fall within the range of antepartum depression reported for Nigeria and sub-Saharan Africa [8, 9, 11, 18]. It is however lower than the prevalence rate reported by a similar study in Ogun State [11] in a cross-sectional study conducted among antenatal care attendees in Ogun state which comprised a population of women with a lower level of education and income. The negative association between education and depression is reported in the literature [7, 17], although this was not confirmed by our study because of the large proportion of educated women (70% having tertiary level education). Variations in the prevalence of APD have been attributed to differences in assessment tools, period of the assessment, methodological issues, and study population [7]. The study mentioned above utilized the EDPS instrument which was used in our study. Besides, our study participants possibly reported their mental health status for a period longer than the seven days stipulated for the EDPS instrument. Hence, some recall bias could account for the lower estimate. In addition, the study population was also different while this current study recruited from comprehensive obstetric facilities within Ibadan, the Ogun state study recruited from the three levels of health care including primary health care centres. Prevalence estimates are crucial for health managers and policy makers for planning, prioritizing and integrating mental health into maternal health care services.

The significant factors associated with antepartum depression on univariate analysis were marital status, employment status, income, history of induced abortion, intimate partner violence, and perceived stress. Studies from different parts of Africa including Nigeria have reported factors such as age, marital status, income, occupation, history of the previous mental disorder, unplanned pregnancy, pregnancy complications, marital dysfunction, and social support as associated with antenatal depression [7, 11, 14, 18]. Generally, the factors associated with antepartum depression are broadly classified into socio-demographic, obstetric and psychosocial factors which were also captured in our study [34]. Being currently married, a proxy variable for partner support was associated with reduced odds of having APD (OR = 0.6) compared with those who were unmarried. Pregnancy is both physiologically and psychologically stressful, hence, partner support is crucial in providing emotional and financial assistance needed to handle pregnancy associated challenges. Many researchers have also supported the role of male partners in alleviating the burden associated with pregnancy [35]. Contrariwise, experiencing intimate partner violence, although uncommon among our study participants, is also inimical to maternal mental health as it increased the risk of APD by four fold in this study. Being employed and having a higher income, both measures of socio-economic status and social determinants of health, lowered the likelihood of APD. Notably, of all the obstetric factors explored only the previous history of induced abortion increased the likelihood of APD. The relationship between previous induced abortion and antepartum depression have been hardly explored by Nigerian researchers. Termination of pregnancy has been described as a stressful life-event that can be accompanied by a wide range of emotional responses which include sadness, grief, loss, regret, anger, a feeling of isolation and so on. These negative feelings may be severe and persistent imposing prolonged psychological burden on women [36, 37].

Multivariate analysis showed high income and perceived stress as significant factors associated with APD, after adjusting for confounders. Actually, perceived stress had a dose–response association with APD which suggests causality. The causal relationship between stress and depression has been reported by researchers [38–41]. Perceived stress during pregnancy could result from low socio-economic status, financial distress particularly the difficulty with meeting basic needs such as food and shelter, lack of social and family support, marital dysfunction and conflict [41, 42]. These factors, together with other early life stressors as physical, sexual and psychological maltreatment could contribute to maternal depression during pregnancy [42]. Physiologically, stressful events activate the hypothalamic–pituitary–adrenal (HPA) and sympathetic nervous responses causing the release of stress hormones like corticotrophin releasing hormones, cortisol, and adrenaline. If these life stressors become prolonged it leads to maladaptive changes that results in mood and anxiety disorders [39]. Ensuring supportive relationship among pregnant women, as well as healthy lifestyle habits such as regular exercise, adequate sleep, avoiding smoking, alcohol and binge eating can be helpful in relieving stress. In this study, the physical activity level was inadequate (26.3 (± 22.9) minutes per week of moderate intensity physical activity compared with the 150 min recommended by WHO [43]. However, there was no difference women with APD and the controls. Physical activity ought to be promoted among pregnancy because of its several benefits which include enhancing the mood by the release of neurotransmitters like endorphins that make people feel good [44]. Importantly, healthy lifestyle habits which are modifiable factors of mental health should be emphasized in maternal health care. For example, we found that binge eating, an eating disorder increased in a dose response fashion among depressed women in our study population. If this disorder is undetected and unchecked it would result in excessive weight gain, postpartum weight retention and obesity in the future [45].

Antepartum depression has been associated with adverse perinatal outcomes such as low birth weight, preterm and intrauterine growth retardation, although these finding have not been consistent [19, 27, 46]. Addressing adverse perinatal outcomes associated with maternal mental health is crucial in neonatal mortality reduction in LMIC. In this study perinatal outcomes examined were low birth weight, macrosomia, preterm delivery, birth asphyxia at 1 min and caesarean section. Only preterm delivery had a significant relative risk with APD (RR = 1.68). The other perinatal outcomes investigated in the study had no significant relationship. Similarly, Dadi and coworkers (2020) in a systematic review and meta-analysis examining, antepartum depression and its association with adverse birth outcomes in low and middle-income countries found the risk of preterm birth and low birth weight was higher in depressed mothers compared to mothers without depression. The biologically plausible reasons for the association between APD and preterm delivery are: the high levels of circulating stress hormones like cortisol [47] which may interfere with placental function causing hypo-perfusion to the feotus and reduce immunity thereby increasing the risk of infection particularly reproductive tract infections. Antepartum depression may also be associated with poor self-care practices in pregnancy such poor nutrition, poor person hygiene that could trigger factors associated with premature contractions [46].

The main strength of our study is the use of a prospective cohort study design which allowed the investigation of multiple perinatal outcomes associated with APD with minimal temporality bias. The study also examined the multiple risk factors associated APD as well as the potential confounders, for example socio-demographic, obstetric, psychological factors hence it was possible to adjust for potential confounders. The use of multiple health facilities also enhanced the generalizability of the study. Our study also highlighted the influence lifestyle characteristics and binge eating on ADP therefore providing empirical evidence for promoting health lifestyle habits to improve maternal mental health so as to optimize pregnancy and neonatal outcomes.

However, our study also has limitations. First is the bias from losses to follow up which is typical of prospective cohort studies, but was accounted for by assuming a 40% attrition in the sample size calculation. The self-reported assessment of APD using the EPDS is subject to measurement error from under-reporting and recall bias. However, the EPDS is a valid and useful instrument in screening for depression in late pregnancy among Nigerian women [29] with EPDS ≥ 12 for major depression at sensitivity = 1.000, specificity = 0.961. Also residual confounding from unmeasured variables could account for some of our findings. The study also recommends further research on the models of integrating mental health into maternal health services.

## Conclusions

APD was prevalent among our study population. The significant factors identified in this study can be targeted to reduce the occurrence of APD among pregnant women in Nigeria through appropriate health interventions which includes APD screening, counselling, and the provision of support for pregnant women during antenatal care as well as lifestyle modification.

## Data Availability

The Ibadan Pregnancy Cohort Study is an ongoing study it is premature to put the data in the public domain now. Besides the datasets generated contain potentially identifying and confidential information. However, data sharing could be considered at a later time on reasonable request from the corresponding author if they meet the criteria for accessing confidential data.
